# JP3 enhances the toxicity of cisplatin on drug-resistant gastric cancer cells while reducing the damage to normal cells

**DOI:** 10.7150/jca.50306

**Published:** 2021-01-30

**Authors:** Yi Zhang, Junjie Chen, Zhen Che, Chuanjun Shu, Dongyin Chen, Kun Ding, Aiping Li, Jianwei Zhou

**Affiliations:** 1 Department of Molecular Cell Biology & Toxicology, Center for Global Health, School of Public Health, Nanjing Medical University, Nanjing 211166, China.; 2 The Key Laboratory of Modern Toxicology, Ministry of Education, School of Public Health, Nanjing Medical University, Nanjing 211166, Jiangsu, China.; 3 Department of Bioinformatics, School of Biomedical Engineering and Informatics, Nanjing Medical University, Nanjing 211166, China.; 4 Department of Medicinal Chemistry, School of Pharmacy, Nanjing Medical University, Nanjing 211166, China.

**Keywords:** Gastric cancer, cisplatin resistance, JP3 polypeptide, synergistic and detoxifying effect.

## Abstract

**Background:** Cisplatin (DDP) is a highly effective chemotherapeutic agent to most solid tumors including gastric cancer (GC), however, its clinical value is limited due to severe toxic side effects and secondary drug resistance. JP3, a JWA protein based MMP2-targeted polypeptide, known to inhibit the growth of GC *in vivo*. However, the bidirectional effects of JP3 in DDP-resistant GC and normal cells have not been demonstrated. The present study aims to investigate the actions of JP3 on protecting normal cells from the toxicity of DDP while enhancing its anti-tumor effects on GC cells.

**Methods:** Routine laboratory experimental methods including CCK-8 assay, Western blotting, Hoechst staining, immunofluorescence (IF) and qRT-PCR were used in mechanism investigation; protein docking analysis and coimmunoprecipitation (Co-IP) were used for prediction and confirmation of interactions between JP3 and CK2. Mouse xenograft model was used for screening the treatment of JP3 plus DDP on GC growth.

**Results:** DDP showed similar toxicities to normal cells and DDP-resistant GC cells; JP3 competitively inhibited the binding of XRCC1 to CK2, reduced the DNA repair and anti-apoptosis capacity of DDP-resistant GC cells in combination with DDP treatment; meanwhile, JP3 protected normal cells from DDP-induced oxidative stress and DNA damage through ERK/Nrf2 signaling. JP3 combined with DDP showed similar bidirectional effects *in vivo*.

**Conclusions:** JP3 enhanced the inhibitory effects of DDP on tumor growth while reduced toxic side effects of DDP on normal cells. The results of this study provide a new insight for the treatment of drug-resistant GC.

## Introduction

Gastric cancer (GC) is the fifth commonly diagnosed cancer worldwide and third leading cause of cancer death 1. In China, gastric cancer ranked first (13.6%) among diagnosed cancers of the digestive tract, second (13.5%) among diagnosed cancers in men and fifth (7.1%) among women 2. Currently, the major treatment for GC is chemotherapy plus surgical removal 3. Cisplatin (DDP) is one of the first-line chemotherapeutic agents for GC. The limitations of DDP include drug resistance in cancer cells and toxicity to non-target tissues which hinder its clinical application 4. The widely accepted mode of action for DDP is binding to cellular DNA and form DNA-DDP adduct, resulting in preventing DNA replication until the damage is repaired 5. However, increased DNA repair in cancer cells is a main cause of DDP resistance 6; and XRCC1 is a vital factor in single strand break repair (SSB) and base excision repair (BER) which causes the development of DDP resistance in various cancers 7. Meanwhile, the toxicity of DDP including neurotoxicity, ototoxicity and gastrointestinal toxicity 8 have been attributed to various reasons which are, in turn, linked with reactive oxygen species (ROS) mediated oxidative stress 9. Therefore, identification and development of novel agents that can sensitize GC cells to DDP and reduce damages to normal cells would have significant impact upon DDP-based chemotherapy.

JWA, also known as ARL6IP5, was initially cloned from human tracheal bronchial epithelial cells after treatment with all-trans retinoic acid 10. Our previous data showed that in DDP-resistant GC cells, JWA down suppresses XRCC1 expression via CK2—p-XRCC1 signaling and enhances DDP-induced DNA damage and apoptosis 11; while, in normal cells, JWA is involved in cellular responses to oxidative stresses and works as a DNA repair protein 12. To find anticancer peptides that mimic JWA, we have recently designed and obtained an optimized polypeptide JP3. Our data showed that JP3 exerts therapeutic roles on GC through inhibition of angiogenesis via the TRIM25-SP1-MMP2 signaling 13. Herein, we investigated the potential bidirectional roles and mechanisms of JP3 on DDP induced toxicity on drug-resistant GC cells and normal cells.

## Materials and methods

### Cell cultures and reagents

Human embryonic kidney cells HEK293T, human gastric epithelial cells GES-1, GC cell lines BGC823 and SGC7901 were purchased from the Type Culture Collection of the Chinese Academy of Sciences (Shanghai, China). We obtained the BGC323/DDP, SGC7901/DDP lines as described previously 14. The BGC823, SGC7901, BGC323/DDP, SGC7901/DDP and GES-1 cells were cultured in RPMI-1640 medium. The HEK293T cells were cultured in DMEM medium. All the cell lines were cultured with 100 U/ml penicillin and 100 μg/ml streptomycin and 10% fetal bovine serum. Cisplatin was obtained from Sigma-Aldrich (St. Louis, MO, USA). JP3 (peptide sequence: Ac-RMKKRYPTT(p)FVMVVGGGGHWGF-NH2; peptide purity: > 98.0%) was synthesized by contracted service of Zhejiang Peptides Biotech Co., Ltd. (Hangzhou, Zhejiang, China).

### Cytotoxicity assay

BGC823, SGC7901, BGC823/DDP, SGC7901/DDP, GES-1 and HEK293T cells were plated at a density of 5000 cells per well in 96-well plates. On the next day, cells were treated with cisplatin at indicated concentrations. Cell Counting Kit-8 (CCK-8) was used to determine cell viability after 24 h treatments according to the manufacturer's instructions (Dojindo, Kumamoto, Japan). The cell variable curves were plotted and the IC_50_ values were evaluated by non-linear regression analysis with Graph Pad Prism software (La Jolla, CA, USA). The cell survival rates are expressed as the means ± S.D. from at least three independent experiments.

### Apoptosis assay

Apoptosis was determined by Hoechst 33342 staining (Beyotime Institute of Biotechnology, China). Cells were seeded in 12-well plates. After treatment with the indicated concentrations of cisplatin and/or JP3 for 24 h, the cells were fixed with 4% paraformaldehyde and then stained with the Hoechst 33342 solutions for 15 min at 37℃. Images were acquired under a Nikon fluorescence microscope. Uniformly stained nuclei were scored as healthy, viable cells. Condensed or fragmented nuclei were scored as apoptotic cells.

### Western blotting

Whole cell lysates were prepared with RIPA lysis buffer containing a protease inhibitor cocktail (Roche, Manheim, Germany). Western blotting analysis was performed as previously described 15. The following antibodies were used as follows: monoclonal anti-JWA (1:500, contract produced by AbMax, Beijing, China); polyclonal anti-PARP-1, anti-γ-H2AX, anti-XRCC1, anti-Bcl-2, anti-CK2α, anti-CAT, anti-Nrf2, anti-p-XRCC1 and p-ERK (1:1000-2000, Cell Signaling Technology, Danvers, MA, USA). Tubulin and GAPDH were used as loading controls.

### Measurement of GSH-Px, SOD and CAT

Cells were incubated with cisplatin and/or JP3 for 24 h. Glutathione peroxidase (GSH-Px), catalase (CAT), and superoxide dismutase (SOD) activity contents were estimated by the appropriate kits according to the manufacturer's protocols (Nanjing Jiancheng Bioengineering Institute, Nanjing, China). At least three biological replications were conducted for all experiments.

### Immunofluorescence microscopy

After treatments, cells were fixed with 4% paraformaldehyde, followed by incubated with anti-γ-H2AX (Cell Signaling Technology, Danvers, MA, USA) or 8-OHdG antibody (Santa Cruz, Dallas, TX, USA) monoclonal antibodies at a 1:200 dilution overnight at 4° C. Then, the cells were incubated with FITC green-conjugated anti-rabbit IgG or Cy3 red-conjugated anti-mouse secondary antibody (1:400, Beyotime, Jiangsu, China) for 2 h at 37° C. After washing with PBST, the nuclei were counterstained with DAPI (Beyotime, Jiangsu, China) for 15 min. The confocal images of the cells were sequentially acquired with Zeiss AIM software on a ZeissQ7 LSM 700 confocal microscope system (Carl Zeiss Jena, Oberkochen, Germany).

### RNA extraction and reverse transcription-PCR

Total RNA was extracted from the cells using TRIzol reagent (Gibco, Gaithersburg, MD, USA) and 500 ng of RNA was used for a reverse transcription reaction with HiScript Q RT SuperMix for qPCR (Vazyme, Jiangsu, China). The cDNA was amplified with the following primers: 5'- GGAGCGAGATCCCTCCAAAAT -3' (forward) and 5'- GGCTGTTGTCATACTTCTCATGG -3' (reverse) for GAPDH; 5'- TGGAGCTGGTAACCCAGTAGG -3' (forward) and 5'- CCTTTGCCTTGGAGTATTTGGTA -3' (reverse) for Catalase; 5'- CAGTCGGTGTATGCCTTCTCG -3' (forward) and 5'- GAGGGACGCCACATTCTCG -3' (reverse) for GPx; 5'- TCAGCGACGGAAAGAGTATGA -3' (forward) and 5'-CCACTGGTTTCTGACTGGATGT -3' (reverse) for Nrf2; 5'- GGTGGAATGGGGAAATCAAGAT -3' (forward) and 5'- TGATGATGTTGGGACCTCCTC -3' (reverse) for CK2α. GAPDH was used as the internal reference, and the results for each sample were normalized to GAPDH expression.

### Immunoprecipitation

The cell extracts were centrifuged at 12, 000×g at 4 °C for 15 min, and the supernatant was divided into two parts. One part was used for western blot, the remaining protein (500 μg) had anti-XRCC1 antibody or IgG antibody (2 μg, Beyotime, Jiangsu, China) added and was cultured in 4 °C for 1 h, and then incubated with Protein A/G agarose beads overnight. Afterwards, the beads were collected by centrifugation and washed four times at 1, 000×g for 5 min at 4 °C with precooling IP buffer. The immunoprecipitate was collected by centrifugation and resuspended in 1 × SDS loading buffer and examined by western blot at last.

### Protein docking analysis

I-TASSER (Iterative Threading Assembly Refinement) was designed for protein structure model by iterative threading assembly simulations. The molecular structures of CK2, JP3 and XRCC1 and the CK2α possible binding site for JP3 or XRCC1 were constructed by I-TASSER.

### BGC/DDP cells xenograft mice model

Male BALB/c nude mice, 4-5 weeks old, weight 20-25 g were purchased from Model Animal Research Center of Nanjing University (Nanjing, China). The animal study proposal was approved by the Animal Care Committee of Nanjing Medical University, Nanjing, China. The mice were housed in a specific pathogen-free (SPF) environment (temperature 23±2° C, 12:12 h light/dark cycle and humidity 50±10% with free access to standard food and water.

After acclimation for one week, the mice were subcutaneously injected with BGC823/DDP GC cells (5 × 10^6^) and when the average tumor volume reached 100-150 mm^3^ the mice were randomly divided into 4 groups including vehicle group, DDP group (4 mg/kg every 3 days), JP3 group (25 mg/kg, Bid) and cisplatin+JP3 group. The mice body weight and tumor volume were measured every 3 days. Serum biochemical indicators were determined with an automatic biochemical analyzer according to the manufacturer's operating instructions. Pathological examinations of the major organs were performed by routine H&E staining.

### Statistics analysis

All statistical analyses were performed with GraphPad Prism 6 software and/or SPSS 20.0. The data were expressed as the means ± S.D. The differences among multiple groups were analyzed by one-way-ANOVA, and the differences between two groups were calculated using parametric unpaired Student's t test. *P* < 0.05 was considered statistically significant.

## Results

### DDP shows similar toxicities to normal cells and drug-resistant GC cells

**Figure 1:**
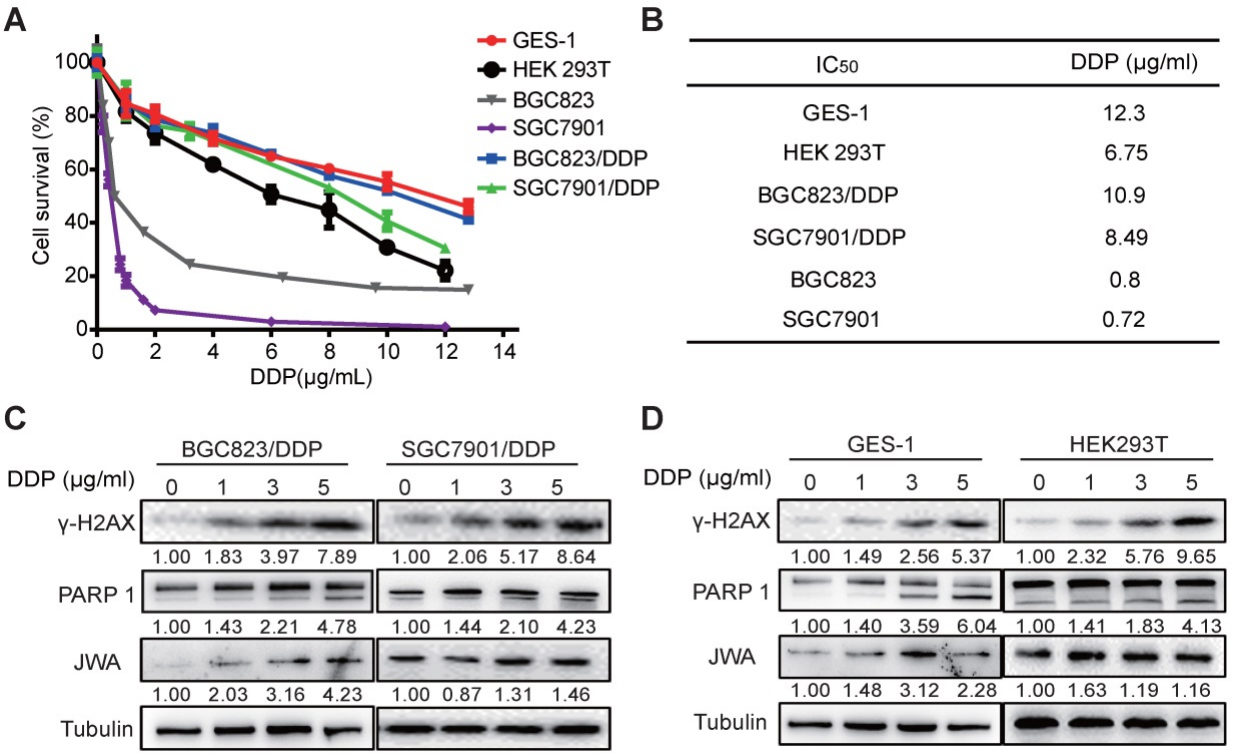
** DDP shows similar toxicities to normal cells and cisplatin-resistant GC cells.** (A) IC_50_ values of DDP in the indicated cell lines were determined. GES-1, HEK293T, BGC823, SGC7901, BGC823/DDP and SGC7901/DDP cells were treated with DDP (0-12 μg/ml) for 24 h. (B) IC_50_ values were calculated based on the results of cell viability measured by CCK-8. (C) BGC823/DPP, SGC7901/DDP, and (D) GES-1, HEK293T cells were treated with DDP at 0, 1, 3, 5 μg/ml for 24 h. The protein levels of γ-H2AX, cleaved PARP-1 and JWA were detected by western blotting. Tubulin was used as the loading control. The intensities of protein bands were analyzed by densitometry after normalization to the corresponding tubulin levels.

To understand the cytotoxicity of DDP on normal and cancer cells, we first evaluated the IC_50_ values of DDP by CCK-8 assay in six cell lines including normal cells (GES-1 and HEK 293T), GC cells (BGC823 and SGC7901), and DDP resistant GC cells (BGC823/DDP and SGC7901/DDP). As shown in Figure 1A, the survival rates of the cells were reduced in a dose-dependent manner after treatment with DDP at the indicated dose for 24 h. The IC_50_ values of BGC823/DDP (10.9 μg/ml) and SGC7901/DDP (8.49 μg/ml) were significantly higher than their parental DDP-sensitive GC cells (0.8 μg/ml for BGC823, 0.72 μg/ml for SGC7901) (Figure 1B); the resistance index (RI) of BGC823/DDP and SGC7901/DDP were 13.63 and 11.79, respectively. Importantly, although the IC_50_ value was higher in GES-1 (12.3 μg/ml) than that in DDP-resistant GC cells, it was obviously lower in HEK293T (6.75 μg/ml) than in DDP-resistant GC cells. To confirm the cytotoxicity of DDP on the cells, both DNA damage and apoptosis biomarkers were determined. The results showed that the expressions of both γ-H2AX and cleaved PARP1 in DDP-resistant GC cells and normal cells were dose-dependently increased after exposure of DDP at 0, 1, 3 and 5μg/ml for 24 h (Figure 1C-D). JWA expressions in these cells also showed a slight increase after DDP exposure. These results suggest that compared with cisplatin resistant GC cells, DDP showed similar toxicities to both normal cells. The toxicity of DDP to HEK293T cells was even greater than that to cisplatin resistant GC cells.

### JP3 plays a bidirectional role in DDP treated GC and normal cells

**Figure 2:**
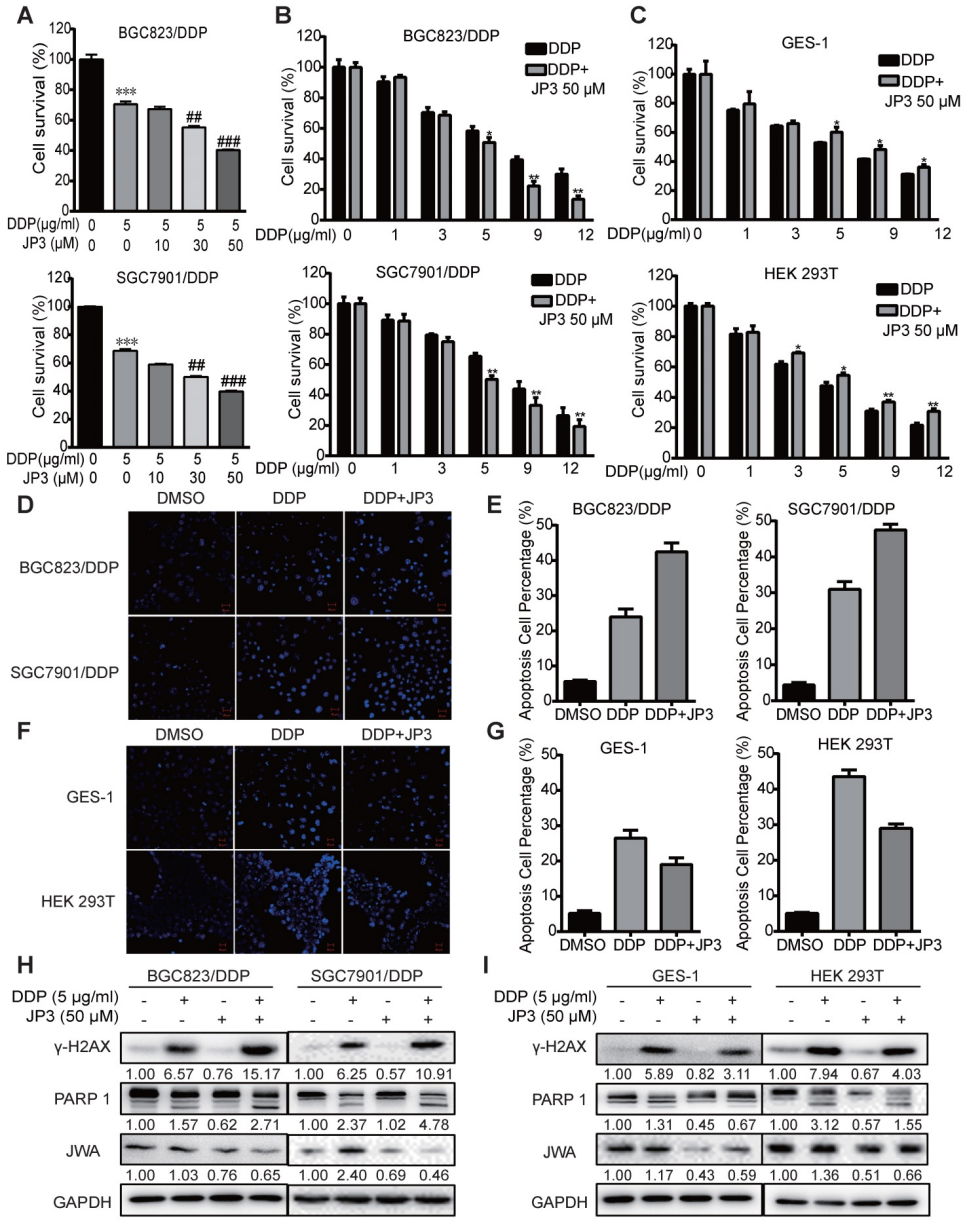
** JP3 plays bidirectional roles in DDP treated GC and normal cells.** (A) BGC823/DDP and SGC7901/DDP cells were treated with 5 μg/ml DDP and the indicated doses of JP3 for 24 h; (B) BGC823/DPP, SGC7901/DDP, and (C) GES-1, HEK293T cells were treated with 50 μM JP3 with or without different doses of DDP for 24 h. The cell viability were measured by CCK-8 assay. BGC823/DDP, SGC7901/DDP (D, E) and GES-1, HEK293T cells (F, G) were treated with DMSO, 5 μg/ml DDP or 50 μM JP3 plus 5 μg/ml DDP for 24 h, and the Hoechst staining images showed cell apoptosis. Scale bars = 100 μm. Quantitative data of apoptosis ratios of (D, F) were shown in (E, G), respectively. BGC823/DDP, SGC7901/DDP (H) and GES-1, HEK293T cells (I) were treated with DMSO, 5 μg/ml DDP, 50μM JP3 or DDP+JP3 for 24 h. Protein levels of γ-H2AX, cleaved PARP-1 and JWA were determined by western blot. GAPDH was used as the loading control.

JP3 is a functional phosphorylated fragment of JWA protein and linked with HWGF for targeting MMP2. To clarify whether JP3 exerted differential roles in DDP treated GC and normal cells, we completed cytotoxicity assays for the treatment of cisplatin in combination with JP3. Both BGC823/DDP (Figure 2A) and SGC7901/DDP (Figure 2B) cells were treated with either a fixed dose of DDP 5 μg/ml combined with a different dose of JP3 (0, 10, 30, 50 μg/ml), or a fixed dose of JP3 (50 μg/ml) combined with a different dose of DDP (1, 3, 5, 9, 12 μg/ml) for 24 h. The data showed that DDP induced a dose-dependent reducing in cell survival rates; however, the cell survival rates in JP3 combined with DDP treatment reduced more obvious than that in DDP exposure alone. These results suggest that JP3 dose-dependently enhanced the toxicity of DDP in both BGC823/DDP and SGC7901/DDP GC cells. The similar assay was conducted in GES-1 and HEK293T normal cells. As shown in Figure 2C, DDP treatment alone induced a dose-dependent toxicity in both cells; very interestingly, however, JP3 combined with DDP treatment significantly increased cell survival rates in both cells. The results suggested that JP3 partly protected normal cells from DDP induced damage.

To confirm the above data, we completed Hoechst assays. As shown in Figure 2D-E, compared to DDP treatment alone, JP3 enhanced pro-apoptotic toxicity of DDP in GC cells; in contrast, JP3 reduced pro-apoptotic toxicity of DDP in normal cells (Figure 2F-G). Further, the expressions of biomarkers indicated that DDP alone induced increasing protein levels of γ-H2AX and cleaved PARP1; and these effects were further enhanced by JP3 in GC cells (Figure 2H). However the expressions of γ-H2AX and cleaved PARP1 were reduced by JP3 in normal cells (Figure 2I). In addition, JP3 also reduced the JWA expression levels in both GC and normal cells (Figure 2H-I). These results suggest that JP3 plays bidirectional effects in DDP treated GC and normal cells, respectively.

### JP3 down-regulates CK2 expression and reduces XRCC1-mediated DNA repair in DDP-resistant gastric cancer cells

**Figure 3:**
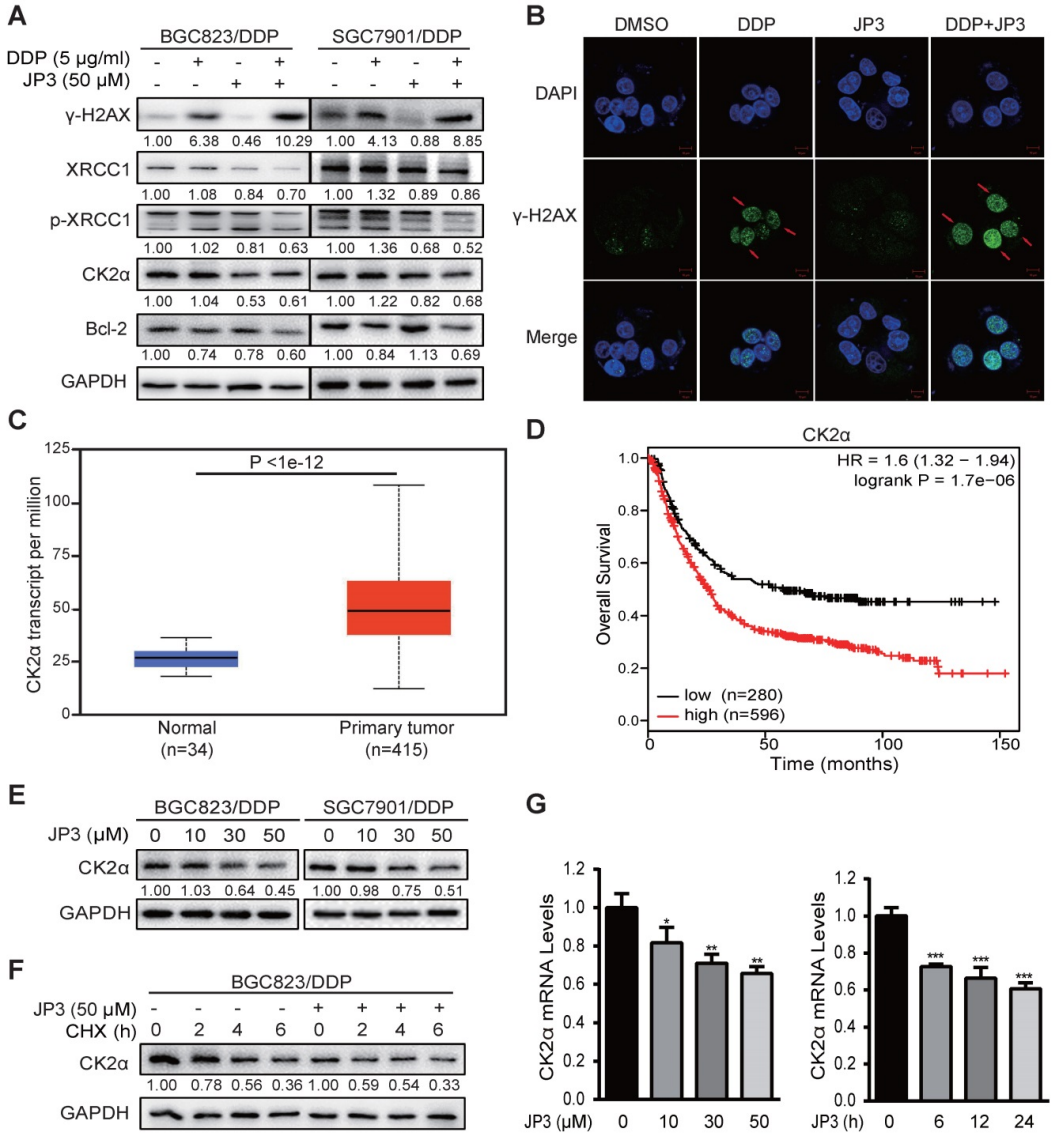
**JP3 down-regulates CK2 expression and reduces XRCC1-mediated DNA repair in DDP-resistant gastric cancer cells.** (A) BGC823/DDP and SGC7901/DDP cells were treated with DMSO, 5 μg/ml DDP, 50 μM JP3 or DDP+JP3 for 24 h. Protein levels of γ-H2AX, XRCC1, p-XRCC1, CK2α and Bcl-2 were determined by western blot. GAPDH was used as the loading control. (B) BGC823/DDP cells were treated with DMSO, 5 μg/ml DDP, 50 μM JP3 or DDP+JP3 for 24 h and immunofluorescence images showed γ-H2AX foci staining. Scale bars = 20 μm. (C) Expression of CK2α mRNA in normal and primary GC tumor tissues were shown. (D) Kaplan-Meier curves depicting OS according to the expression patterns of CK2α in GC cohort. *P* values were calculated with the log-rank test. (E) BGC823/DDP and SGC7901/DDP cells were treated with 0, 10, 30, 50 μM JP3 for 24 h, respectively. Protein levels of CK2α were determined by western blot. GAPDH was used as the loading control. (F) BGC823/DDP cells were treated with DMSO or 50 μM JP3 for 24 h and then exposed to cycloheximide (CHX) at 50 μg/ml for the indicated times. CK2α protein stability was determined by western blot. (G) BGC823/DDP cells were treated with 0, 10, 30, 50 μM JP3 for 24 h or 50 μM JP3 for 0, 6, 12, 24 h; and then the mRNA levels of CK2α were evaluated by qPCR assay. Error bars indicate the means ± S.D. *P < 0.05, **P < 0.01, ***P < 0.001.

Our previous data showed that JWA down-regulates XRCC1 via ubiquitination of CK2, therefore reverses the resistance of GC cells 11. Here, we determined if JP3 worked in GC cells through similar mechanism of JWA gene. As shown in Figure 3A, compared to DDP treatment alone, JP3 in combination with DDP significantly increased γ-H2AX level in DDP-resistant GC cells; on the contrary, expressions of XRCC1/p-XRCC1, CK2α and Bcl-2 were down-regulated in DDP-resistant GC cells. The immunofluorescence confocal assay also showed increased γ-H2AX foci in BGC823/DDP cells after treatment with JP3 plus DDP (Figure 3B), the quantified fluorescence intensity was shown in Figure S1A. The TCGA database showed that expression of CK2α was higher in 415 primary GC tumor samples than in 34 normal samples (Figure 3C, http://ualcan.path.uab.edu/). We further constructed Kaplan-Meier survival curves (http://kmplot.com/analysis/) and found that the patients with high CK2α expression had poor OS compared with low CK2α ones (*P* < 0.01; Figure 3D). Western blotting data showed the expression levels of CK2α were decreased by JP3 in dose dependent manner (Figure 3E). Moreover, CK2α was degraded more rapidly in JP3-treated BGC823/DDP cells than in control cells (Figure 3F). Treatment of BGC823/DDP cells with MG132 increased the expression of CK2α; however, MG132 did not show effects on CK2α by JP3 treatment (Figure S1C). To determine how JP3 reduced CK2α expression, we firstly completed RT-PCR assays. Data showed that JP3 dose/time-dependently down-regulated CK2α expression in BGC823/DDP cells (Figure 3G).

### JP3 competitively inhibits interactions between CK2α and XRCC1 in DDP-resistant GC cells

**Figure 4:**
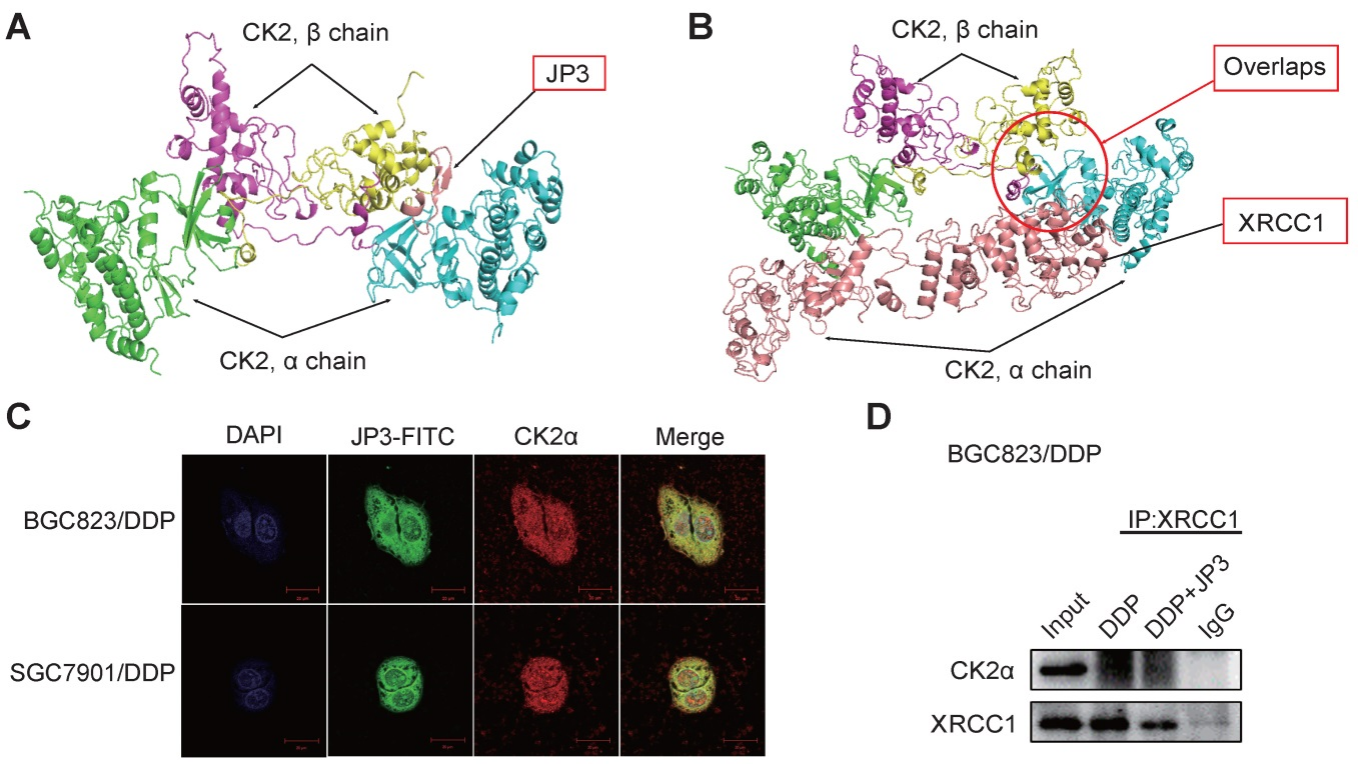
** JP3 competitively inhibits interactions between CK2α and XRCC1 in DDP-resistant GC cells.** (A, B) Protein-protein interaction molecular dockings of CK2α and JP3, CK2α and XRCC1 were shown. (C) BGC823/DDP and SGC7901/DDP cells were treated with JP3-FITC for 6 h, and the representative immunofluorescence images showed the co-localizations among JP3-FITC (green), CK2α (red) and DAPI (blue). Scale bars = 20 μm. (D) BGC823/DDP cells were pretreated with DDP or DDP+JP3 for 24 h, and the endogenous protein-protein interaction between CK2α and XRCC1 was determined by co-IP with XRCC1 antibodies followed by western blot confirmation.

Since the phosphorylation of XRCC1 by CK2 is required for its stability and efficient DNA repair in SSB 16. To determine whether JP3 inhibited interactions between CK2α and XRCC1, we completed computational molecular docking analysis. As a result, the interaction between CK2α and JP3 was identified and the binding sites of the both were overlapped with those between XRCC1 and CK2α (Figure 4A-B). Immunofluorescence confocal images further confirmed that the co-localization between JP3 and CK2α was existed in both BGC823/DDP and SGC7901/DDP cells (Figure 4C). These results were also supported by co-IP assay in Figure 4D, Figure S1D, data showed the interaction between XRCC1 and CK2 was weakened in the cells treated by JP3 in combination with DDP compared to the cells treated by DDP alone. Suggesting that JP3 down-regulates expressions of CK2α/XRCC1 through several mechanisms including at least transcriptional and competitive inhibitions.

### JP3 attenuates DDP-induced DNA damage in normal cells by reducing ROS production

**Figure 5:**
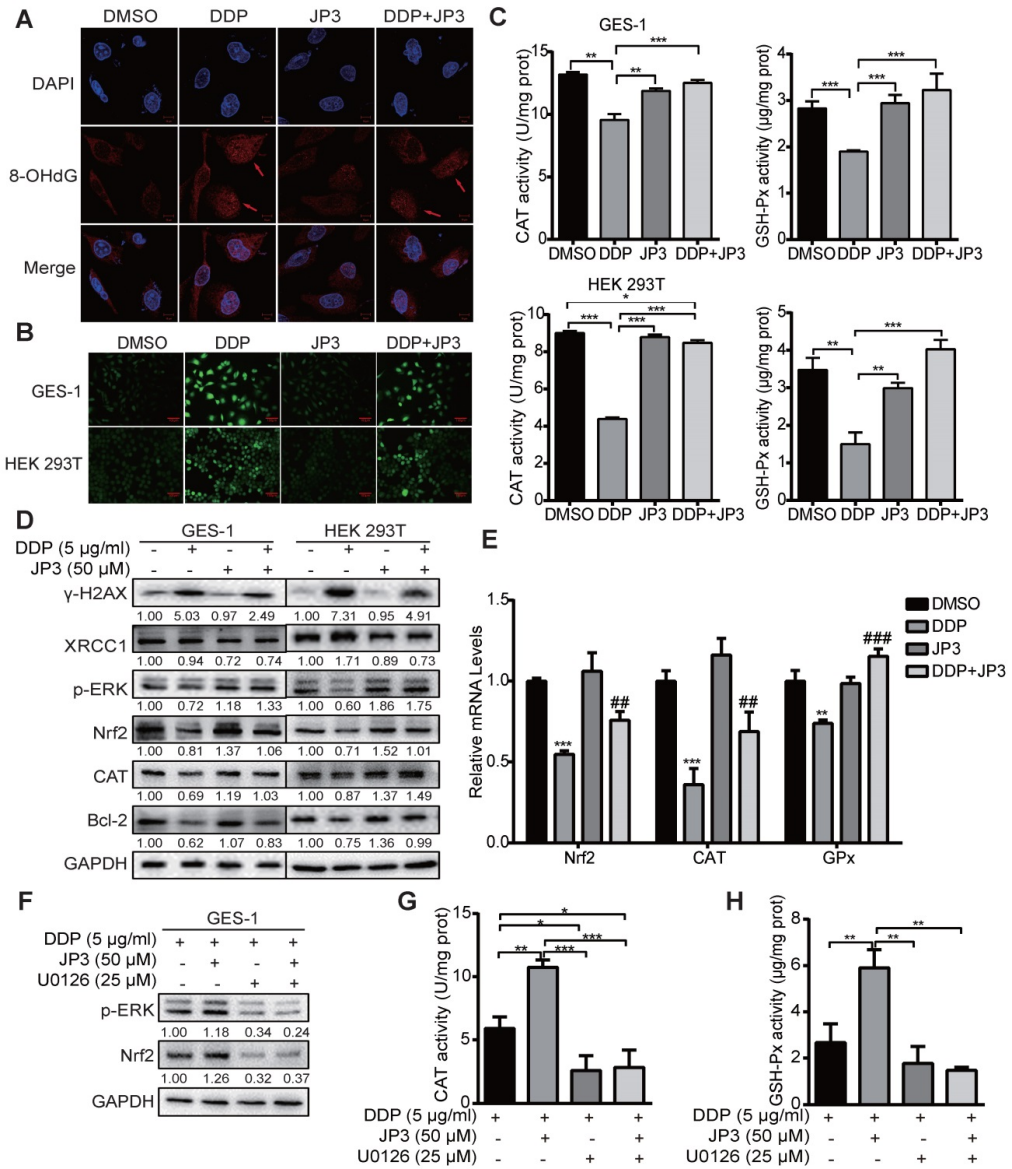
** JP3 attenuates DDP-induced DNA damage in normal cells by reducing ROS.** (A) GES-1 cells were treated with DMSO, 5 μg/ml DDP, 50 μM JP3 or DDP+JP3 for 24 h; the images showed immunofluorescence staining of 8-OHdG (red) and DAPI (blue). Scale bars = 20 μm. (B) GES-1 and HEK 293T cells were treated with DMSO, 5 μg/ml DDP, 50 μM JP3 or DDP+JP3 for 24 h; intracellular ROS levels were measured using a fluorescent dye (DCFH-DA). Scale bars = 100 μm. (C) The content of intracellular activities of CAT and GSH-Px were measured by related commercial kits in both GES-1 and HEK293T cells under indicated treatments. (D) Protein levels of γ-H2AX, XRCC1, p-ERK, Nrf2, CAT and Bcl-2 were determined by western blot under indicated treatments; GAPDH was used as the loading control. (E) The mRNA levels of Nrf2, CAT and GPx in GES-1 cells were evaluated by qPCR assay, and the error bars indicate the means ± S.D. *P < 0.05, **P < 0.01, ***P < 0.001 compared with DMSO, and ^#^*P* < 0.05, ^##^*P* < 0.01, ^###^*P* < 0.001 compared with DDP treatment. (F) GES-1 cells were treated with 5 μg/ml DDP or 50 μM JP3 alone or in combination, and with or without U0126 (25 μg/ml) for 24 h, then the protein levels of p-ERK, Nrf2 were determined by western blot; and activities of CAT and GSH-Px were determined by commercial kits (G-H). For all graphs, error bars indicate the means ± S.D. *P < 0.05, **P < 0.01, ***P < 0.001.

DDP is known to generate cytotoxicity and side effects in patients by oxidative stress and direct DNA damage related events 17. Our previous data showed JWA as a DNA repair protein involves in regulation of oxidative stress and cell response process 18. To determine if the protection roles of JP3 on normal cells from DDP toxicity was through its anti-oxidant mechanism, we conducted related assays. As shown in Figure 5A, JP3 reduced 8-OHdG levels by DDP in GES-1 cells compared with DDP treatment alone. Reactive oxygen species (ROS) also decreased by the treatment of JP3 plus DDP in GES-1 and HEK 293T cells compared with that by DDP alone (Figure 5B); in addition, the numbers of DNA damage foci and DCFH-DA positive cell were also reduced in JP3 plus DDP cells compared to DDP treatment alone. (Figure S2A-B).

To elucidate the underlying mechanisms of JP3 in anti-DDP triggered oxidative stress, the intracellular antioxidant enzymes GSH-Px, CAT and SOD were also analyzed. As shown in Figure 5C, JP3 reversed DDP-induced declines in GSH-Px and CAT activities in GES-1 and HEK393T cells, however, JP3 has no significant role on DDP reduced SOD activity (Figure S2C).

We have previously identified that JWA rescues the accumulation of paraquat generated ROS via MEK/PI3K-Nrf2 signaling 19, here we determined if JP3 was also worked through this signaling. The result showed JP3 activated the phosphorylation of ERK and up-regulated both Nrf2 and CAT expressions and therefore reversed DDP mediated oxidative stresses in both GES-1 and HEK193T cells; in addition, JP3 increased Bcl-2 expressions in the both cells (Figure 5D). As shown in Figure 5E, JP3 also transcriptional rescued DDP reduced expressions of Nrf2, GSH-Px and CAT in GES-1 cells. To confirm whether ERK associated signal pathway involved in anti-oxidant roles of JP3 in the normal cells, U0126 was used to block ERK signaling. As shown in Figure 5F, the pharmacological inhibition of ERK by U0126 abolished the effects of JP3 from DDP induced oxidative stress, and the elevated expression of Nrf2, and GSH-Px and CAT activities by JP3 were also blocked by U0126 (Figure 5G-H). These results suggest that JP3 through activating of ERK/Nrf2 linked anti-oxidant system protected normal cells from DDP induced oxidative stress and DNA damage; JP3 also enhanced anti-apoptosis potential in these cells.

### JP3 exerts bidirectional roles in BGC823/DDP xenografts model mice

**Figure 6:**
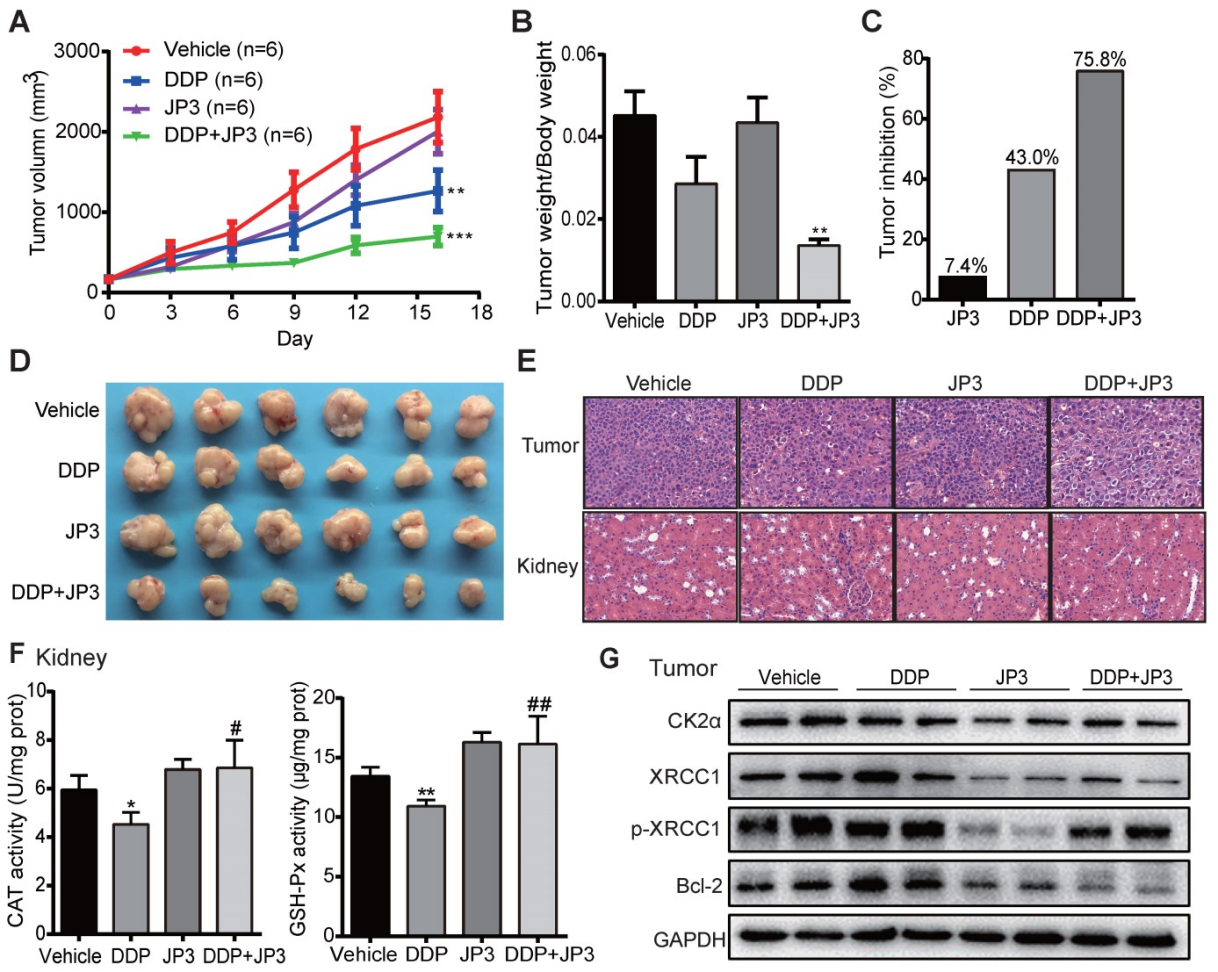
** JP3 exerts bidirectional roles in BGC823/DDP xenografts model mice*.*** (A) The tumor volumes of BGC823/DDP cell xenografts were measured every three days for all experimental mice, n=6; *P < 0.05, **P < 0.01, ***P < 0.001 values were compared with vehicle group. (B) the ratio of tumor weight to body weight and the tumor inhibition rates (C) were determined on day 16 after treatments; (D) the tumor mass images and representative H&E staining images from tumors and kidney sections (original magnification: 200×, scale bar: 50 μm) were shown in (E). (F) The activities of CAT and GSH-Px of kidney tissues from the four groups were determined by related commercial kits, and error bars indicate the means ± S.D. *P < 0.05, **P < 0.01, ***P < 0.001 compared with vehicle, and ^#^*P* < 0.05, ^##^*P* < 0.01, ^###^*P* < 0.001 compared with DDP treatment. (G) The representative protein levels of CK2α, XRCC1, p-XRCC1, CK2α and Bcl-2 in tumor tissues were determined by western blot; GAPDH was used as the loading control.

To verify whether JP3 has a similar bidirectional effect on DDP-treated GC *in vivo*, we established a BGC823/DDP GC cell xenograft mouse model. As shown in Figure 6A, compared with DDP treatment alone, the inhibition of tumor growth in DDP+JP3 group was enhanced obviously. The ratio of tumor/body weight was significantly lower in DDP+JP3 group than that in DDP alone group (Figure 6B). Accordingly, the tumor inhibition rates were 7.4%, 43.0% and 75.8% in JP3, DDP and DDP+JP3 groups compared with the vehicle control group, respectively (Figure 6C-D). Moreover, histological examination revealed that the numbers of apoptotic cell was increased in tumor tissues obviously in DDP+JP3 group compared to that in DDP group; and the inflammatory cell infiltration in kidney tissues were relieved in DDP+JP3 group compared with DDP group (Figure 6E). In addition, the significantly reduced GSH-Px and CAT activity in mouse kidney tissues treated with DDP alone was reversed in mouse treated with JP3 combined with DDP (Figure 6F). Finally, we determined expressions of related biomarkers in tumor tissues. As shown in Figure 6G, DDP treatment increased the protein levels of CK2α, XRCC1, p-XRCC1 and Bcl-2 were suppressed in both JP3 alone and JP3 plus DDP groups. In addition, the results of serum biochemical markers showed that although DDP treatment reduced LDH activities from 1036.3±164.59 U/L in untreated group to 747.5± 131.37 U/L in DDP group, the enzymes of ALT (DDP vs control): 22.8±1.71 vs 18.0±2.94 and BUN (DDP vs control): 11.7±1.70 vs 7.9±1.29; CREA: 21.5±4.20 vs 14.8±0.96) caused by DDP were also obviously. JP3 alone showed a reduction of LDH activity and an obvious protection role on kidney (CREA (JP3 vs control): 11.3±0.96 vs 14.8±0.96); in JP3 plus DDP group, importantly, tumor burdens were further reduced compared to DDP alone group, however, liver and kidney functions were improved (Table S1). These data further confirmed a synergistic effects on cancer cells and a protecting effects on liver and kidney cells by JP3 when combined with DDP.

## Discussion

**Figure 7:**
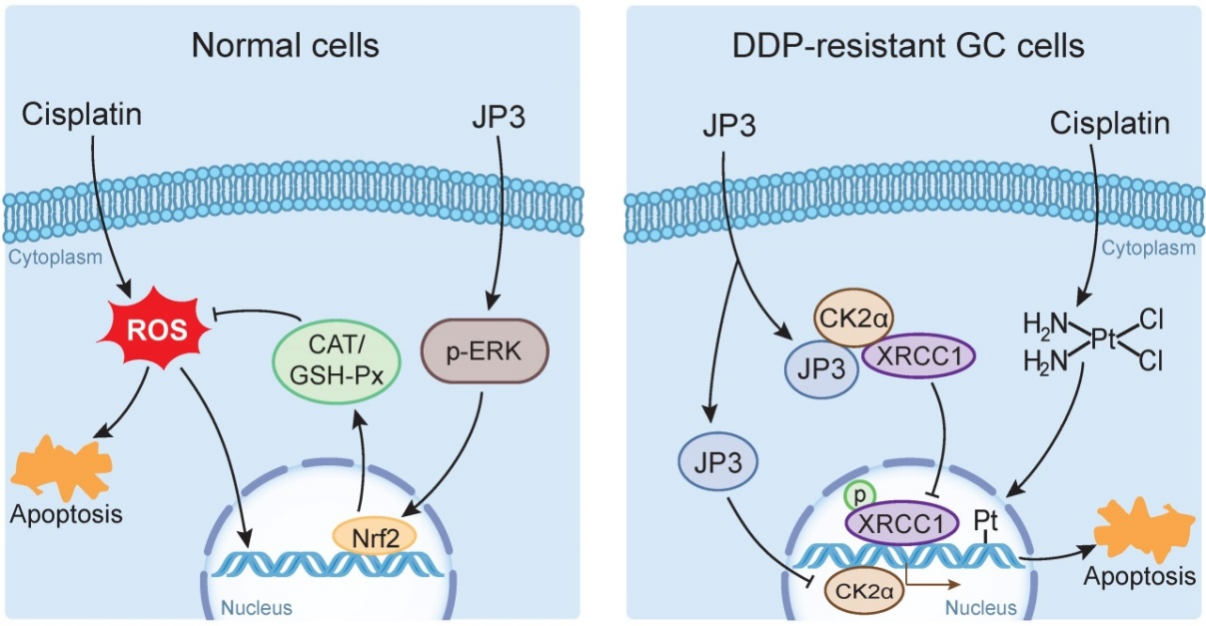
The bidirectional action mode and mechanism of JP3 combined with DDP in GC and normal cells

DDP is a highly effective chemotherapeutic agent used in the treatment of solid tumors including GC, but its toxic side effects and secondary resistance are still two obvious challenges in the clinic 20. In this study, we reported for the first time that although JP3 alone showed a weak suppression on DDP-resistant GC cells proliferation *in vivo*, it exerted bidirectional roles when used in combination with DDP. On the one hand, JP3 restored the sensitivity of the DDP resistant GC cells; on the other hand, JP3 reduced toxic side effects of DDP in model mice. The mechanism of action preliminarily elucidated in this study includes that JP3 weakened the DNA repair and anti-apoptosis ability of GC cells through CK2α-XRCC1 signaling pathway. JP3 also enhanced the antioxidant capacity of mouse, thus reduced the toxic and side effects of DDP (Figure 7).

Tumor cells generate DDP resistance by enhancing DNA damage repair capabilities 21; XRCC1 plays an important role in the single strand break repair (SSB) and base excision repair (BER) 22, 23. Expressions of XRCC1 and JWA protein in GC tissue is reported a prognostic and predictive role 24, and overexpression of JWA leads to the inhibition of CK2—p-XRCC1—XRCC1 pathway 11. Our current results revealed several novel connection and roles of JP3 in DDP-resistant GC. CK2 is highly expressed in a variety of cancers and is associated with tumor growth 25, it also synergistically works with other oncogenes 26, 27; several CK2 inhibitors have been reported to be effective in reducing tumor resistance 28, 29. In this study, CK2α expressions was down-regulated in mRNA transcription level by JP3 with or without DDP in time-dose dependent manner. It is worth pointing out that JP3 reduced activation of XRCC1 by competitively binding with CK2α and thus CK2α unable to activate XRCC1. Previously, we demonstrated that JWA accelerated the degradation of CK2α through the ubiquitination mediated mechanism, thus reduced the activation of XRCC1 11. Therefore, although JP3 and JWA protein could reduce XRCC1 activity through CK2α, their molecular mechanisms were completely different.

It is well known that DDP treatment induces a series of side effects including neurotoxicity, ototoxicity and gastro toxicity 30. DDP is able to shift the redox balance in cells by conjugation leading to ROS over production 31, and therefore oxidative stress is the main mechanism underlying DDP-induced toxicity 32, 33. JWA is involved in cellular responses to suppress environmental stress including oxidative stress 12; JWA also protects neuronal cells from damage after exposure to the environmental toxicant PQ by inhibiting ROS production and increases glutathione content 19. In the present study, GES-1 and HEK293T cells exposed to DDP underwent serious oxidative stress caused-DNA damage. JP3 combined with DDP treatment declined ROS by activating ERK and Nrf2, a key factor in oxidative stress response 34, and increasing expression levels of antioxidant enzymes, therefore protected cells from DDP induced toxicity.

Elevated copper levels is a well-documented metabolic difference between malignant cells and 'normal' cells 35, 36. Rizvi A et al. have demonstrated that calcitriol, the metabolically active form of vitamin D, interacted with copper and produced reactive oxygen species by Fenton-Haber-Weiss like reactions which caused irreparable DNA damage in carcinoma cells and induced the consequent selective cell death of malignant cells but spare normal cells. The mechanism of calcitriol-copper interaction has been confirmed in malignancy like rabbit lymphocytes 37, human peripheral blood lymphocytes 38 and hepatocellular carcinoma rat modal 39. It has been previously shown that cisplatin also interact with cellular copper, and this interact may in part, be responsible for its chemotherapeutic effects 40. Whether the selective toxicity of JP3 is related to the difference of copper levels between normal and tumor cells requires further investigation. Our results showed that JP3 may prevent oxidative damage from DDP by scavenging and clearing reactive oxygen species. Whether JP3 affects enzymatic and non-enzymatic scavengers of reactive oxygen species in cancer cells or not needs more experiments.

Elevated copper levels are a well-documented metabolic difference between malignant cells and 'normal' cells 35, 36. Rizvi A et al. have demonstrated that calcitriol, the metabolically active form of vitamin D, interacted with copper and produced reactive oxygen species by Fenton-Haber-Weiss like reactions which caused irreparable DNA damage in carcinoma cells and induced the consequent selective cell death of malignant cells but spare normal cells. The mechanism of calcitriol-copper interaction has been confirmed in malignancy like rabbit lymphocytes 37, human peripheral blood lymphocytes 38 and hepatocellular carcinoma rat modal 39. It has been previously shown that cisplatin also interact with cellular copper, and this interact may in part, be responsible for its chemotherapeutic effects 40. Whether the selective toxicity of JP3 is related to the difference of copper levels between normal and tumor cells requires further investigation. Our results showed that JP3 may prevent oxidative damage from DDP by scavenging and clearing reactive oxygen species. Whether JP3 affects enzymatic and non-enzymatic scavengers of reactive oxygen species in cancer cells or not needs more experiments.

The bidirectional effects of JP3 have also been demonstrated *in vivo* models. The efficacy of JP3 combined with DDP significantly decreased tumor growth compared to the monotherapy. Furthermore, the combined treatment also inhibited the expression of CK2α, p-XRCC1, XRCC1 and the anti-apoptosis marker Bcl-2 41. Bcl-2 is known as an important anti-apoptotic gene, which can inhibit cell apoptosis and prolong cell survival. Many studies have confirmed that cisplatin resistance may relate with the high expression of Bcl-2 in tumor cells 41, 42. In our study, JP3 treatment reduced the expression of Bcl-2 in DDP-resistant tissues and cells, but increased the expression of Bcl-2 in normal cells. Bcl-2 may also be a key point in the bidirectional effects of JP3, the specific mechanism needs further research. Our studies also showed that the inflammation and injury of kidney were relieved by enhancement of GSH-Px and CAT activities. The evidences in the present study indicate that JP3 in combination with DDP could be used as a potential treatment for reducing DDP resistance in GC cells and toxic side effects in mouse. Whether JP3 has a similar effect in combination with other chemotherapeutic drugs that produce oxidative stress or DNA damage needs further study. Since this study was conducted using cell culture models and cell lines based xenograft mouse models, the bidirectional effects of JP3 in combination with DDP need to be further validated in PDX models and other solid tumors. The potential value of JP3 as a single new anticancer drug also needs further evaluation.

Polypeptides, characterized by low molecular weight, high activity, low toxicity and easy modification, are widely concerned in anti-cancer experiments and clinical applications 43, 44. JP3 is a pre-phosphorylated and MMP2-targeted 22 amino acids polypeptide, which simulates some biological functions of JWA protein. JP3 plays an important role in reducing cisplatin tolerance on GC cells and toxic side effects in mice. Our study may provide new insights for cisplatin in the treatment of drug-resistant gastric cancer.

## Supplementary Material

Supplementary figures and tables.

## Abbreviations

DDP: cisplatin
GC: gastric cancer
CK2: casein kinase 2
Nrf2: nuclear factor erythroid 2-related factor 2
XRCC1: X-ray repair cross complementing group1
SSB: single strand break
BER: base excision repair
ROS: reactive oxygen species
JWA: ADP-ribosylation-like factor 6 interacting protein 5
PARP1: poly(ADP-ribose) polymerase 1
γ-H2AX: phosphorylated histone H2AX
DAPI: 4' 6-diamidino-2-phenylindole dihydrochloride
CHX: cycloheximide
CAT: catalase
SOD: superoxide dismutase
GPx: glutathione peroxidase
DCFH-DA: 2',7'-dichlorodihydrofluorescein diacetate
IC_50_: half maximal inhibitory concentration
