# Supplementary material for: JP3 enhances the toxicity of cisplatin on drug-resistant gastric cancer cells while reducing the damage to normal cells
Source: J Cancer. 2021 Jan 30;12(7):1894–906. doi: 10.7150/jca.50306 (PMC7974513; doi:10.7150/jca.50306)
Supplement: Supplementary file 1 — Supplementary figures and tables. [file jcav12p1894s1.pdf]

Figure S1. JP3 increases DNA damage in DDP-resistant GC cells by down regulating CK2α expression and competitively inhibiting the interaction between CK2α and XRCC1. (A) BGC823/DDP cells were treated with DMSO, 5 μg/ml DDP, 50 μM JP3 or DDP+JP3 for 24 h and γ-H2AX foci staining was quantized by Image J. (B) BGC823/DDP cells were treated with DMSO or 50 μM JP3 for 24 h and with or without 10 μM MG132 for 6 h. CK2α protein stability was determined by western blot; GAPDH was used as the loading control. (C) SGC7901/DDP cells were pretreated with DDP or DDP+JP3 for 24 h, and the endogenous protein-protein interaction between CK2α and XRCC1 was determined by co-IP with XRCC1 antibodies followed by western blot confirmation.

Figure S2. JP3 attenuates DDP-induced DNA damage in normal cells by reducing ROS. (A) GES-1 cells were treated with DMSO, 5 μg/ml DDP, 50 μM JP3 or DDP+JP3 for 24 h; the immunofluorescence staining of 8-OHdG (Figure 4 A, red) was quantized by Image J. (B) GES-1 and HEK 293T cells were treated with DMSO, 5 μg/ml DDP, 50 μM JP3 or DDP+JP3 for 24 h; the numbers of DCFH-DA positive cells (Figure 4B) were calculated. (C) The content of intracellular activity of SOD was measured by related commercial kits.

Table S1. Results of serum biochemical parameters of BGC823/DDP cell xenograft model mice

Figure S1

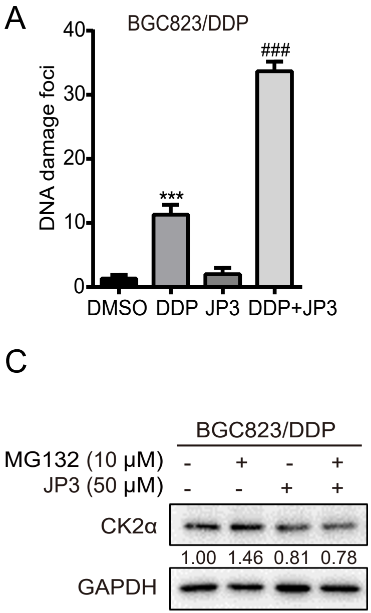
A
BGC823/DDP
DNA damage foci
40
30
20
10
0
***
###
DMSO
DDP
JP3
DDP+JP3
B
BGC823/DDP
SGC7901/DDP
JP3 (h)
0
6
12
24
0
6
12
24
CK2α
1.00
0.66
0.50
0.41
1.00
0.74
0.45
0.33
GAPDH
C
BGC823/DDP
MG132 (10 μM)
- + - +
JP3 (50 μM)
- - + +
CK2α
1.00
1.46
0.81
0.78
GAPDH
D
SGC7901/DDP
IP:XRCC1
Input
DDP
DDP+JP3
IgG
CK2α
XRCC1

Figure S2

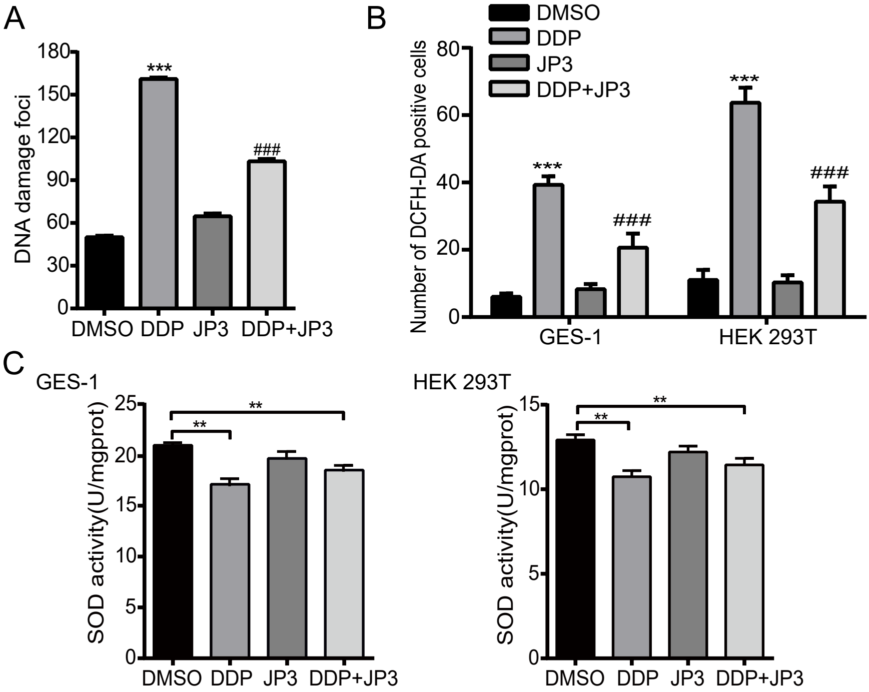
A
DNA damage foci
180
150
120
90
60
30
0
***
###
DMSO
DDP
JP3
DDP+JP3
B
DMSO
DDP
JP3
DDP+JP3
Number of DCFH-DA positive cells
80
60
40
20
0
***
###
***
###
GES-1
HEK 293T
C
GES-1
SOD activity(U/mgprot)
25
20
15
10
5
0
**
**
DMSO
DDP
JP3
DDP+JP3
HEK 293T
SOD activity(U/mgprot)
15
10
5
0
**
**
DMSO
DDP
JP3
DDP+JP3

Serum Index	Groups			
	Vehicle	DDP	JP3	DDP+JP3
ALT (U/L)	18.0±2.94	22.8±1.71*	18.5±1.91	20.5±2.38
AST (U/L)	68.3±9.98	84.5±17.33	69.8±12.71	81.3±20.09
TP (g/L)	31.2±5.34	43.0±5.79*	31.6±5.80	38.18±1.39*
ALB (g/L)	18.6±3.23	27.2±3.79*	20.7±2.82	25.5±1.67*
BUN (mmol/L)	7.9±1.29	11.7±1.70*	7.0±0.81	11.5±1.07*
CREA (μmol/L)	14.8±0.96	21.5±4.20*	11.3±0.96**	17.3±3.30
LDH (U/L)	1036.3±164.59	747.5±131.37*	925.8±292.94	647.3±161.29*
CK-MB (U/L)	278.5±56.88	258.0±68.82	244.8±62.96	252.3±69.71
CK (U/L)	702.8±135.70	995.0±525.48	606.5±202.70	934.5±297.45

*P < 0.05, **P < 0.01 (vs. Vehicle); N=4
